# Comparison of RNN-LSTM, TFDF and stacking model approach for weather forecasting in Bangladesh using historical data from 1963 to 2022

**DOI:** 10.1371/journal.pone.0310446

**Published:** 2024-09-19

**Authors:** Md. Mahmudul Hasan, Md. Jahid Hasan, Parisha Binte Rahman

**Affiliations:** 1 Department of Mechanical and Production Engineering (MPE), Islamic University of Technology (IUT), Board Bazar, Gazipur, Bangladesh; 2 Department of Civil Engineering, Ahsanullah University of Science and Technology, Dhaka, Bangladesh; Atlantic Technological University, IRELAND

## Abstract

Forecasting the weather in an area characterized by erratic weather patterns and unpredictable climate change is a challenging endeavour. The weather is classified as a non-linear system since it is influenced by various factors that contribute to climate change, such as humidity, average temperature, sea level pressure, and rainfall. A reliable forecasting system is crucial in several industries, including transportation, agriculture, tourism, & development. This study showcases the effectiveness of data mining, meteorological analysis, and machine learning techniques such as RNN-LSTM, TensorFlow Decision Forest (TFDF), and model stacking (including ElasticNet, GradientBoost, KRR, and Lasso) in improving the precision and dependability of weather forecasting. The stacking model strategy entails aggregating multiple base models into a meta-model to address issues of overfitting and underfitting, hence improving the accuracy of the prediction model. To carry out the study, a comprehensive 60-year meteorological record from Bangladesh was gathered, encompassing data on rainfall, humidity, average temperature, and sea level pressure. The results of this study suggest that the stacking average model outperforms the TFDF and RNN-LSTM models in predicting average temperature. The stacking average model achieves an RMSLE of 1.3002, which is a 10.906% improvement compared to the TFDF model. It is worth noting that the TFDF model had previously outperformed the RNN-LSTM model. The performance of the individual stacking model is not as impressive as that of the average model, with the validation results being better in TFDF.

## 1. Introduction

The global impact of climate change is being experienced worldwide, rendering it one of the paramount issues of the twenty-first century. Precipitation variation is a clear and measurable consequence of climate change that can negatively impact the ecology, socioeconomic conditions, and public health of a region. Weather-dependent technology has posed considerable challenges for Transmission and Distribution System Operators (ISO and USU professionals) in terms of balancing and integrating with the grid [1]. This emphasizes the significance of utilizing the most accurate forecasting methods that are accessible. Weather forecasting research has flourished in the past decade and has almost reached the same level of importance as wind and load forecasting, as shown by the numerous publications on the subject [2–5].

### 1.1. Statistical trends of machine learning in weather prediction

In recent years, artificial intelligence has caused a significant change in the fields of weather forecasting and trend prediction [6–12]. Latif et al. [13] worked on comparing different major predictive models for rainfall prediction using machine learning, statistical methods, and deep learning. They found that LSTM models demonstrated superior performance for predicting rainfall. The evaluation was based on RMSE, R^2^, and MAE measures, and the study highlighted the potential of remote sensing and hybrid predictive models for further investigation. Ding et al. [14] worked on improving city-scale air temperature mapping for understanding urban heat islands using predictive models. They found that regression kriging combined with multiple linear regression performed best, with an RMSE of 0.92°C and an R^2^ of 0.959. Their model can reduce the number of weather stations in Guangzhou by 50% while maintaining accuracy. Bian et al. [15] worked on improving the accuracy of runoff prediction by developing an integrated data-driven model combining Long Short-Term Memory (LSTM) and Light Gradient Boosting Machine (LightGBM). They found that the LSTM-LightGBM model outperformed the single models, achieving an NSE of 0.92, RMSE of 0.075 million m^3^, and MAE of 0.046 million m^3^. Ayoub et al. [16] worked on developing statistical emulators for the Weather Research and Forecasting (WRF) model to enable rapid predictions of wind and temperature fields for nuclear accident response. They found that both deep neural network and vector autoregressive model with exogenous variables (VARX) emulators could forecast wind fields in real-time without sacrificing accuracy, with the autoregressive model particularly excelling in temperature field prediction. Baig et al. [17] investigated the efficacy of advanced machine learning techniques for enhancing rainfall prediction in the hyper-arid environment of the UAE using a 30-year dataset. They found that initial univariate models, particularly XGBoost and ensemble methods, performed well in training (Correlation Coefficient of 0.88) but poorly in testing (Correlation Coefficient of 0.45). Rabindiran et al. [18] examined the impact of air pollution in Chennai to predict the Air Quality Index (AQI) using machine learning models like XGBoost, Random Forest, BaggingRegressor, and LGBMRegressor with historical data from 2017 to 2022. They found that the XGBoost model performed best, achieving an R^2^ of 0.9935, MAE of 0.02, MSE of 0.001, and RMSE of 0.04, while the LightGBM model had a lower R^2^ of 0.9748. The study highlighted that PM2.5 had the greatest impact on AQI (value of 0.91) and noted an increasing AQI trend over the last two years, indicating worsening air pollution if current conditions persist. Moosavi et al. [19] used machine learning techniques to reduce uncertainty in WRF model precipitation forecasts by examining interactions between physical processes and forecast errors. They validated that Random Forests and Artificial Neural Networks can effectively estimate model errors and identify influential physical processes. Jiang et al. [20] proposed the Granular Sigmoid Extreme Learning Machine (GSELM) algorithm by combining a random sigmoid function with the extreme learning machine for improved weather forecasting. They found that GSELM, tested on UCI and Australian weather datasets, predicts next-day weather more accurately and quickly. Dhillon et al. [21] analyzed historical data to quantify maize and soybean yield variability in Ohio and studied the impact of climate change scenarios using machine learning. They found that the Random Forest model performed best, with an RMSE of 0.61 Mt/ha (R^2^ = 0.73) for maize and 0.21 Mt/ha (R^2^ = 0.64) for soybean, predicting significant yield drops by 2100 under both lower and higher emissions scenarios. Akbarian et al. [22] evaluated 1- to 3-month runoff forecasts using ECMWF ensembles over 30 basins in Iran. They found that ANN, XGBoost, and RF models performed best, with KGE’ values of 0.70, 0.68, and 0.66, respectively, while all models showed decreased performance with increased lead times. ANN and XGBoost had the highest accuracy for 2- and 3-month lead times, achieving KGE’ values of 0.65 and 0.60.

### 1.2. Correlation between climate change and machine learning

Dhaka, the capital city of Bangladesh, has a population of over 21 million and serves as the country’s economic and cultural center. It is also one of the fastest-growing cities globally. However, the metropolis is extremely susceptible to the impacts of climate change, especially changes in rainfall patterns. The frequency and intensity of heavy rainfall in Dhaka have escalated, leading to infrastructure damage, landslides, and flooding. Azad et al. [23] proposed hybrid machine learning models, EEMD-ANN and EEMD-SVM, for predicting thunderstorm frequency (TSF) in Bangladesh across high, moderate, and low TSF months. The EEMD-ANN model showed the highest precision for high TSF prediction, while EEMD-SVM excelled for moderate and low TSF predictions, outperforming conventional models by 8.02–22.48% in RMSE. Azmain et al. [24] developed a machine learning-based rainfall prediction system for Bangladesh using Random Forest Regression on weather data from 1901 to 2015. They achieved 91% accuracy by tuning the model, and compared it to the base model, Randomized Search CV, using evaluation metrics like MAE, MSLE, and RMSLE. The results indicate that machine learning can significantly enhance weather forecasting accuracy, contributing to disaster prevention and safety measures. Tahsin et al. [25] analyzed and predicted daily weather patterns in Chittagong city of Bangladesh, using 20 years of weather data and 12 different Data Mining models, categorized into rules-based, tree-based, and function-based. Evaluating the models with metrics like precision, recall, accuracy, F-measure, and ROC area, the study found that the J48 classifier performed the best, achieving an accuracy of 82.30%, precision of 82.40%, recall of 82.20%, f-measure of 84.20%, and a ROC area of 97.8%. Nunno et al. [26] developed reliable precipitation prediction models using machine learning for the tropical monsoon-climate northern region of Bangladesh, focusing on Rangpur and Sylhet. They used M5P and support vector regression (SVR) algorithms and created a hybrid model combining both. The hybrid M5P-SVR model achieved the best predictions, with R^2^ values of 0.87 and 0.92 for Rangpur and Sylhet stations, respectively. Riya et al. [27] proposed a rainfall prediction model using machine learning with weather datasets, focusing on eight features, including temperature, humidity, and solar radiation. Their study demonstrated that the Random Forest classifier achieved the highest accuracy of 87.68% for daily rainfall projections, based on data from 2016 to 2019. They suggest that increasing the data volume to at least ten years could further improve accuracy. Chadee et al. [28] examined the impact of climate change on rainfall trends in Trinidad and Tobago from 1990–2020. Using data from two meteorological stations, the study found no significant trends in annual, seasonal, or decadal rainfall over the 30 years. This suggested that the region is safe from abrupt changes due to climate change and emphasizes the importance of localized climate studies for policy and adaptation strategies. However, the results did not correlate rainfall intensity with negative impacts on the drainage system. Balkissoon et al. [29] used a triangulated grid to estimate areal precipitation in Trinidad, using rain gauge stations as nodal points. The grid was optimized for better rainfall patterns and the average areal precipitation was found to be between 1000 and 3800 mm with an annual aerial rainfall of 2368.8 mm. Niazkar et al. [30] highlighted the ML applications in evaluating climate change effects on water quantity, quality, extreme events, and water supply systems, emphasizing the importance of localized climate studies and novel downscaling techniques for accurate predictions and effective management. Azamathulla et al. [31] presented two studies using ANN and gene expression programming (GEP) to predict atmospheric temperature in the Tabuk region of Saudi Arabia. Results showed the GEP model outperforms the ANN model, making it robust and useful for practitioners. Mampitiya et al. [32] investigated the prediction of PM10 levels in the urbanized regions of Battaramulla and Kandy in Sri Lanka. Five models were evaluated and the LightBGM algorithm demonstrated superior performance in Kandy and Battaramulla. The models were applicable for the purpose of organizing activities in the industrialized area of Battaramulla and the sacred place of Kandy.

The main goal of this research is to make use of the existing meteorological data from Bangladesh in order to forecast the weather. The model employs innovative techniques, specifically RNN-LSTM, TFDF and Stacked Model, which are state-of-the-art in the field of machine learning analysis, notably in the domain of meteorological analysis. The algorithm is a cutting-edge tool in meteorological analysis, utilizing advanced procedures and methodologies to provide accurate and reliable outcomes. The tool’s ability to handle intricate data sets and provide excellent precision renders it a crucial device for understanding weather patterns and improving prediction abilities. This algorithm represents a pivotal shift in meteorological research, enabling the exploration of more sophisticated and streamlined methods for researching atmospheric phenomena.

The application of Machine Learning in weather prediction offers substantial benefits over traditional methods, making it a powerful tool for enhancing the accuracy, efficiency, and relevance of forecasts. By leveraging Machine Learning ’s ability to handle complex relationships, integrate diverse data sources, process information in real-time, and continuously learn and adapt, more precise and actionable weather predictions can be achieved. These advancements not only improve the understanding of weather patterns but also provide critical insights for mitigating the impacts of climate change and extreme weather events. This advancement shows the rationale behind the use of ML in this study and underscores its potential to revolutionize weather forecasting for the scientific community and various industries.

## 2. Methodology

The aim of this study is to create an RNN-LSTM, TFDF, and Stacking Model in order to examine the influence of climate conditions in Bangladesh. This part will discuss the recommended stacking model and the development of the prediction model utilizing the stacking model, XGBoost and LightGBM. In addition, the study will elucidate the process of selecting the base models and meta-models.

### 2.1 Study of data processing

Cleaning up data in the meteorological domain involves resolving issues related to missing values, inaccuracies, and inconsistencies in real-world situations. Various approaches have been devised to address this challenge, and the choice of the appropriate methodology depends on several factors.

Feature scaling is an essential preprocessing step in machine learning that has a major impact on the performance of different algorithms. Algorithms such as TFDF, KNN, SVM and Neural Networks are influenced by the scale of input data, which can be disproportionately impacted by varied scales of certain variables. To optimize the model’s performance and accuracy, it is important to standardize all features to the same scale, thereby avoiding any unfavourable results and improving the overall resilience and precision of the model.

Many machine learning algorithms rely on the magnitude of measurements without considering the particular units of measurement. When features have different ranges, the ones with bigger ranges can have a greater impact on the model’s learning process, which can lead to a skewed outcome. Scaling the features helps to mitigate this issue. In order to ensure uniform scaling across all features, two methods are often used: the Z-score method and the Min-Max scaling algorithm. The Z-score approach standardizes the data by adjusting its mean (μ) to 0 and its standard deviation (σ) to 1, with a particular emphasis on features that are near to zero. This is especially beneficial in algorithms where the computations heavily rely on the mean and variance.

The Min-Max scaling algorithm transforms the characteristics to a predetermined range, usually [0, 1] or [–1, 1], in order to remove the impact of varying scales. This approach is especially beneficial when the data does not conform to a Gaussian distribution or when the values of the features need to be limited within a given range for certain machine learning techniques.

In feature scaling, the initial value of a feature is transformed using $x=\frac{x-\bar{x}}{\sigma }$, known as Z-score standardization. This method centers the data around zero with a standard deviation of 1, ensuring uniform variance.

Another approach is Min-Max scaling, which rescales features to a specified range, such as [0,1] or [−1,1] using $x=\frac{x-xmin}{xmax-xmin}$. This method normalizes features to a common scale, preventing features with larger ranges from dominating the model. Both techniques replace original values with scaled values, enhancing model performance by ensuring feature consistency.

### 2.2 RNN-LSTM

Recurrent Neural Networks (RNNs) are a specific sort of neural network that is specifically intended to process data in a consecutive manner. They possess a concealed state that captures data from preceding inputs, enabling them to represent temporal connections. RNNs are extensively used in tasks such as language modeling, audio recognition, and time series prediction. However, RNNs might encounter difficulties such as vanishing gradients, which can pose challenges throughout the training process for lengthy sequences. Methods such as Long Short-Term Memory (LSTM) and Gated Recurrent Units (GRU) have been created to overcome these restrictions.

LSTM networks are a type of RNN that specifically tackles the issue of the vanishing gradient problem. This characteristic makes them very suitable for processing long sequences of data. Gates are utilized to regulate the flow of information, ensuring the maintenance of long-term dependence. LSTMs are highly proficient at catching intricate temporal patterns and possess the ability to selectively retain or discard information, rendering them highly effective for jobs involving sequential data [33].

In RNN, the decision made at time t—1 has an impact on the decision made at time t. Hence, the network’s response to new data relies on two factors: 1. the present input and 2. the output from the immediate past.

The output of an RNN is computed using the iterative calculation of the following two equations.


$$h_{t}=H(W_{xh}x_{t}+W_{hh}h_{t-1}+b_{h})$$ (1)

$$y_{t}=W_{hy}h_{t}+b_{y}$$ (2)

The specialized memory cell design in LSTM facilitates the storage of information over extended periods. Since then, other individuals have made modifications to the cell structure. However, the traditional formulation of a single LSTM cell can be described by the following equations:

$$f_{t}=\sigma (W_{f}\cdot [h_{t-1},x_{t}]+b_{f}),$$ (3)

$$i_{t}=\sigma (W_{i}\cdot [h_{t-1},x_{t}]+b_{i}),$$ (4)

$${\tilde{C}}_{t}=tanh(W_{C}\cdot [h_{t-1},x_{t}]+b_{C}),$$ (5)

$$C_{t}=f_{t}*C_{t-1}+i_{t}*{\tilde{C}}_{t},$$ (6)

$$o_{t}=\sigma (W_{o}\cdot [h_{t-1},x_{t}]+b_{o}),$$ (7)

$$h_{t}=o_{t}*tanh(C_{t}),$$ (8)

The symbols σ and tanh represent the sigmoid function and the hyperbolic tangent function, respectively. The variables i, f, o, C, and C˜ correspond to the input gate, forget gate, output gate, memory cell content, and new memory cell content, respectively. The sigmoid function is utilized to create three gates within the memory cell, while the tanh function is employed to amplify the output of a specific memory cell.

**Fig 1:**
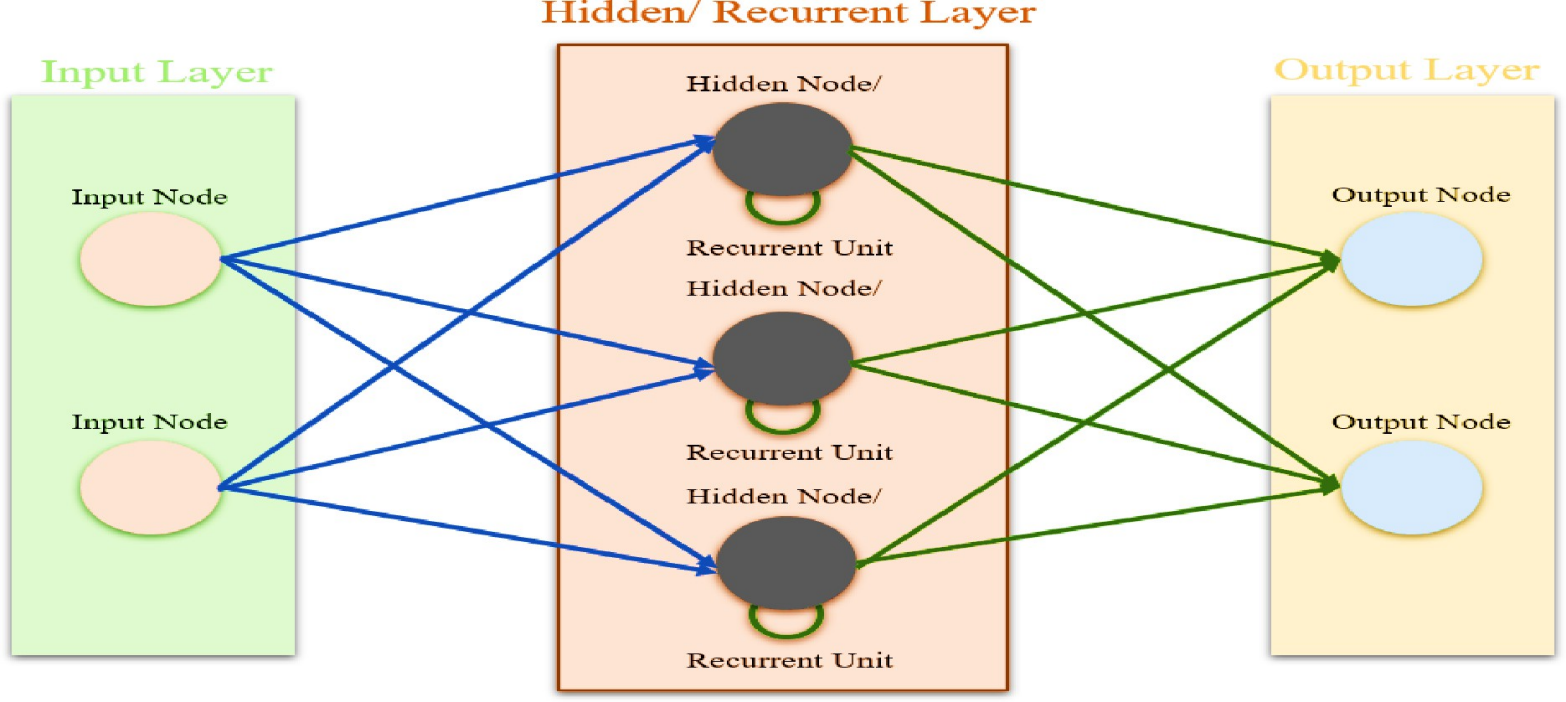
A visual architecture of LSTM-RNN.

**Fig 1** illustrates that the structure of an LSTM comprises an input layer, hidden layers containing LSTM units, and an output layer. The hidden layers of the LSTM network consist of individual units, each of which is equipped with gates (input, forget, and output) that control the flow of information. These gates enable the network to effectively manage and update a cell state as time progresses. The input layer transmits sequential data to the LSTM units, which analyze and retain pertinent information over time steps. The output layer produces the ultimate predictions by utilizing the processed sequences from the hidden layers. This architectural design allows the LSTM to efficiently capture and model long-term dependencies.

Configuration of Parameters:

The runs were conducted with the following parameters:

- The dropout rate is 40%.
- The number of epochs is set to 50.
- The training batch size is set to 20.
- The test batch size is set to 10.
- The activation function used is ReLU.
-The number of neurons in the buried layers is either 500 or 1,000.
- Adam optimizer:
- The value of β1 is 0.99.
- The value of β2 is 0.9999.
- The value of ε is 1e-7.
- The learning rate is set to 0.001.

### 2.3 TensorFlow decision forest

During the model development phase, meteorological data classification neural network models were trained using the Keras TensorFlow Decision Forests (TF-DF) framework. Keras streamlines the creation of deep learning models, facilitating the execution of experiments by seamlessly incorporating deep learning algorithms into applications. Keras enables sophisticated deep learning capabilities within the TensorFlow framework.

**Fig 2:**
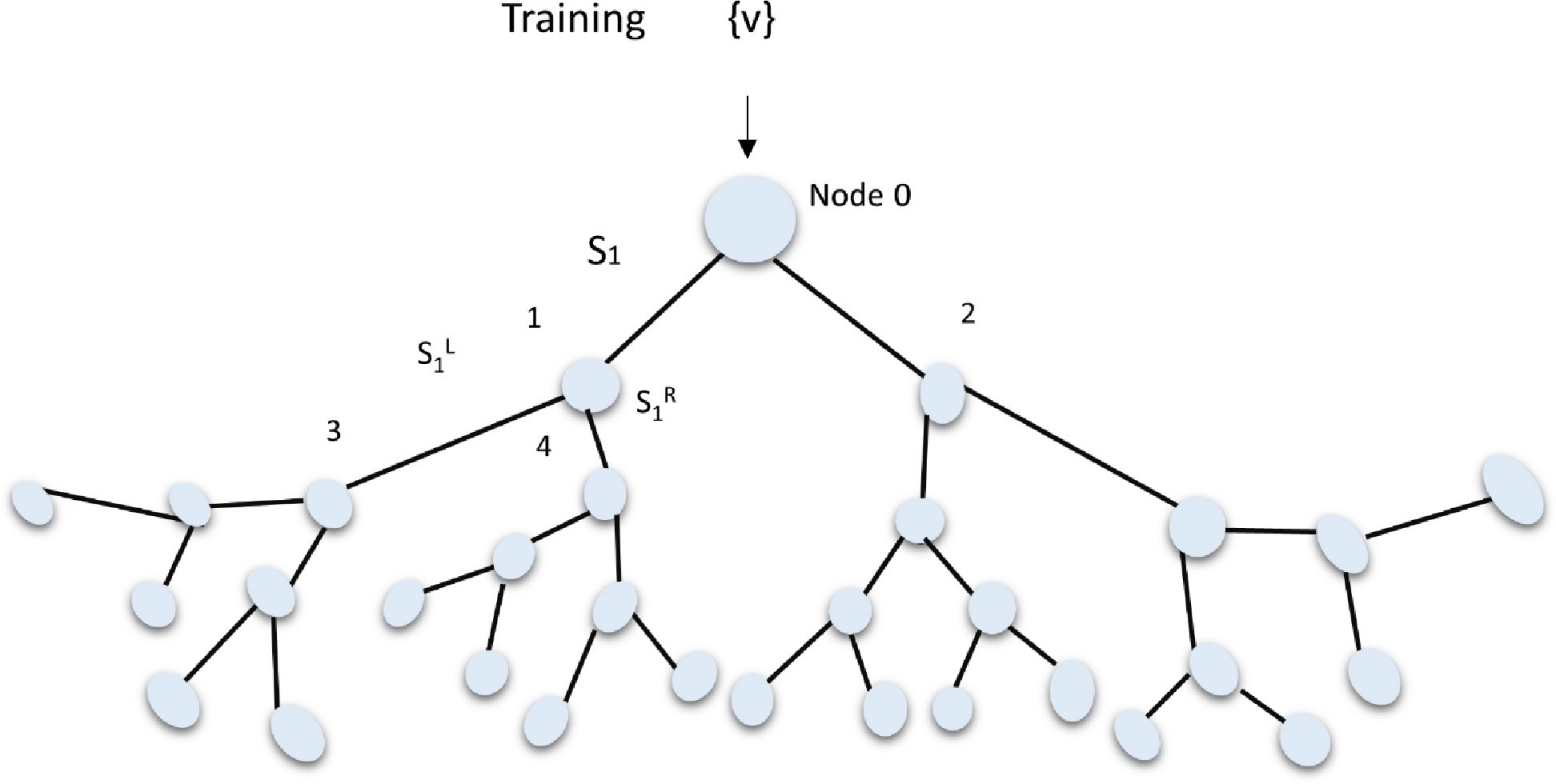
Training of decision tree.

TF-DF is a complete compilation of advanced algorithms specifically developed for the purpose of training, serving, and assessing Decision Forest models. These models have the ability to carry out tasks such as classification, regression, and ranking. They consist of several decision trees that are trained using random techniques. The final forecast of a decision forest is determined by combining the predictions of its component decision trees. A decision tree is a hierarchical arrangement of interconnected nodes. During the training process, the complete dataset, represented as {v}, is fed into the tree. Subsequently, the split nodes are modified in order to optimize a certain energy function, hence improving the precision and efficiency of the model, as depicted in **Fig 2**. The structured methodology enables Decision Forest models to effectively manage intricate tasks by utilizing the combined decision-making of several trees.

**Fig 3:**
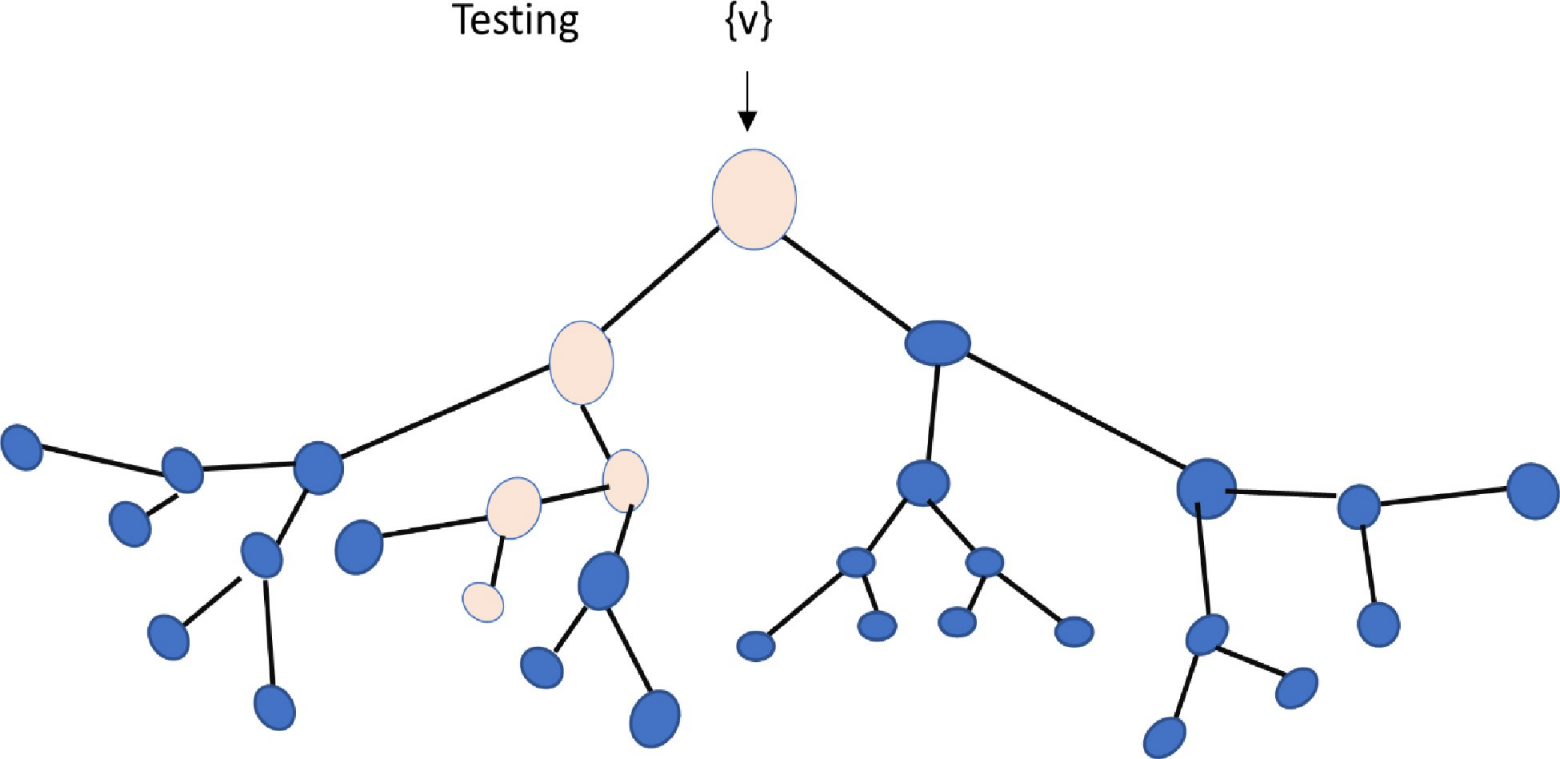
Testing of input data v in a decision tree.

During the testing phase, a split node conducts a test on the input data υ and guides it to the suitable child node, repeating this process recursively until a leaf node is reached, as shown in **Fig 3**.

The application of decision forests in computer vision has yielded remarkable achievements. Recent research has shown that using ensembles of slightly varied trees can result in higher accuracy when applied to unfamiliar and untested data. These trees exhibit exceptional prediction precision while using minimum processing resources. They are capable of handling a wide range of input data and offer effective scalability for huge datasets. This study employed both Gradient-Boosted Trees and Random Forest methods. Boosting is a method that improves the effectiveness of weak learning algorithms by repeatedly applying them to altered versions of the training data, resulting in a notable increase in accuracy. Gradient boosting improves upon this method by training further models to rectify the mistakes made by prior models, thus improving the accuracy of predicting the response variable. Conversely, Random Forests consolidates numerous tree predictors, merging their results to enhance the overall resilience and precision of the model. This combination of approaches exploits the advantages of each algorithm, offering a potent tool for intricate data analysis jobs. Krauss et al. [34] explains that the boosting strategy functions by repeatedly applying weak learners to altered versions of the training data, with the alterations being determined by the weights assigned to each example. Each successive classifier in the boosting process focuses on classifying the cases that posed a challenge in the previous steps. Mathematically, a group of trees can be expressed as:

$${\hat{y}}_{i}=\sum_{k=1}^{K}\;f_{k}(x_{i}),f_{k}\in F$$ (9)

In this case, F is the collection of all possible regression and classification trees, K is the number of trees, and *f*_*k*_ is a function inside the operational domain F. The optimization problem seeks to maximize or minimize the objective function, which is precisely described as:

$$obj\;(\theta )=\sum_{i}^{n}\;l(y_{i},{\hat{y}}_{i})+\sum_{k=1}^{K}\;\omega (f_{k})$$ (10)

where *ω(f*_*k*_*)* represents the computational complexity of the tree *f*_*k*_.

### 2.4 Stacking model

Stacking ensemble learning is a method that merges the advantages of different models to enhance performance and improve generalization capabilities. This approach consists of two separate stages: training the foundational models and training the meta-model. During the initial step, the initial data set is divided into several subsets to be used for both training and testing purposes. The training set is subsequently preprocessed using normal scaling, as depicted in **Fig 9**, to normalize the data and make it suitable for modelling.

In the second phase, the predictions generated by the base models are rearranged in the exact order as the original training data, resulting in the creation of a new training set. The creation of this new training set for the meta-model involves merging the altered training sets from several base models. Similarly, the testing set for the meta-model is created by combining the predictions from the basis models on their individual testing sets. The meta-model is subsequently trained using the freshly compiled dataset.

**Fig 4:**
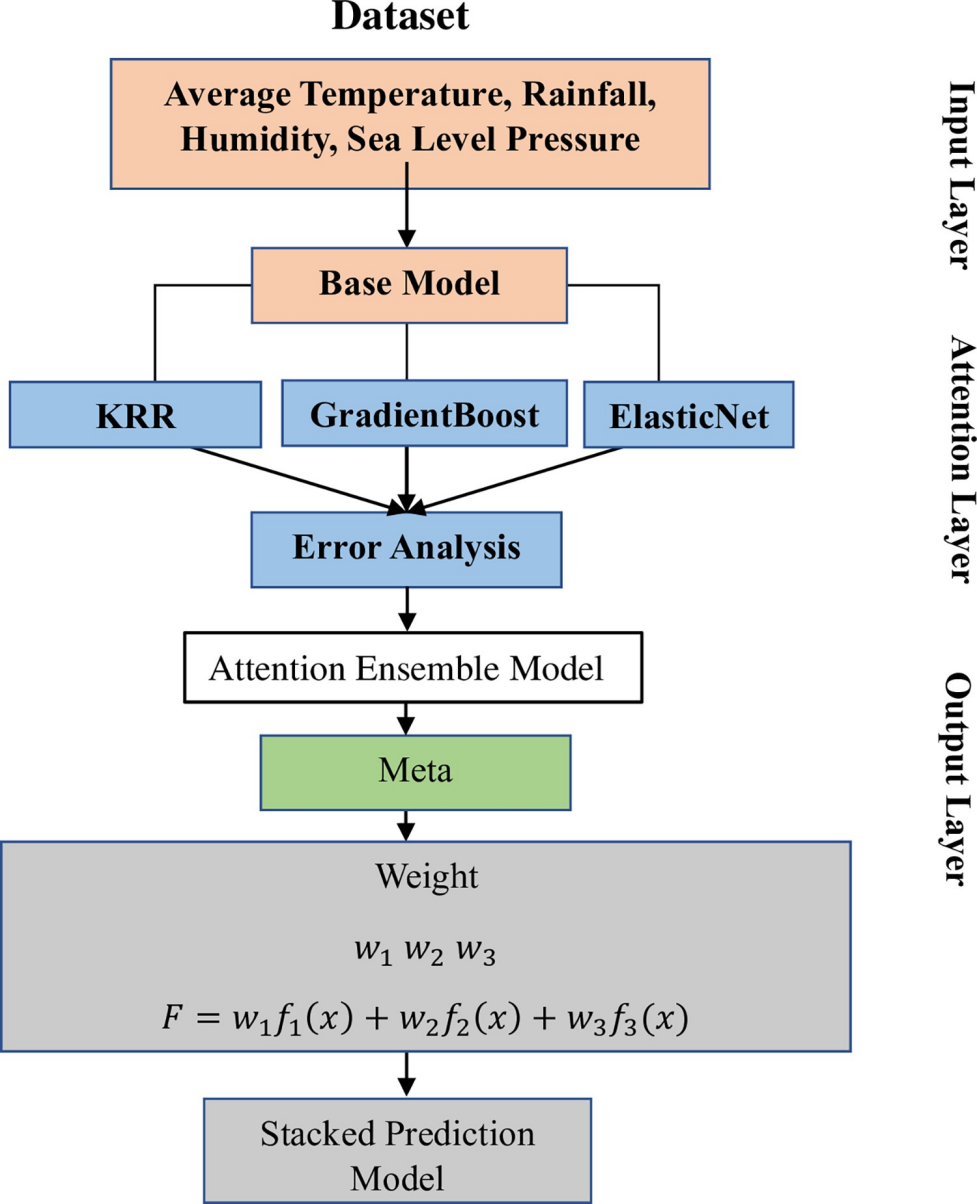
Flowchart depicting the development process of the proposed stacking ensemble model in the current research.

The selection of base models and the meta-model is crucial in stacking ensemble learning. The work involved constructing an ensemble model utilizing three base models: Elastic Net (ENet), Gradient Boosting, and Kernel Ridge Regression (KRR). Furthermore, a meta-model called the attention-based ensemble model (ATE) was created, utilizing the attention mechanism. The ATE model undergoes a series of iterative training steps, as seen in **Fig 4**.

The procedure of generating the proposed attention ensemble model is outlined in the flow chart depicted in **Fig 4**. In addition to the ATE model, the study also utilized basic averaging ensemble and Weighted Averaging Ensemble (WAE) as comparative methodologies to assess the effectiveness of the Attention to Ensemble (ATE) learning strategy. These comparable models serve as a standard for showcasing the efficacy of the ATE model.

The Simple Averaging Ensemble (SAE) approach is derived from the concept of computing the arithmetic mean. Let’s examine an ensemble model comprised of K-base models. The result of the SAE model can be represented in the following manner:

$$f_{SAE}(x)=\frac{1}{K}\sum_{k=1}^{K}\;f_{i}(x),x=1,2,\ldots ,N$$ (11)

Here, *f*_*i*_*(x)* denotes the result of the kth base model, while N represents the overall size of the dataset.

According to the SAE technique, the weights given to the expected values of each base model are equal, which means they do not sufficiently consider the differences in the predictions of each model. The Weighted Averaging Ensemble (WAE) method improves the precision of the ensemble model by merging predictions from many base models, while considering the differences in their prediction accuracy. The result produced through the WAE method can be expressed as:

$$f_{WAE}(x)=\sum_{k=1}^{K}\;\omega_{i}f_{i}(x),x=1,2,\ldots ,N$$ (12)

The weight assigned to the kth base model when the input is x is represented by i(x), and N is the size of the dataset. The value of i(x) is determined by following a specific sequence of steps: First, compute the sum of squared deviations using the expected values for each basic model. Afterwards, calculate the individual weights of each model using Eq 5. The weight can be calculated by doing the computation in the following manner:

$$\omega_{i}(x)=\frac{D_{k}^{-1}(x)}{\sum_{k=1}^{K}D_{k}^{-1}(x)}$$ (13)

### 2.5 Performance evaluation

When evaluating the predictive precision of current models, it is crucial to employ a range of metrics to completely examine mistakes. This approach guarantees a comprehensive assessment of model performance by encompassing many facets of prediction errors. The present study assessed the performance of the models using three specific metrics: Root Mean Square Error (RMSE), Mean Absolute Error (MAE), and Root Mean Squared Logarithmic Error (RMSLE).

RMSE and MAE are often used metrics to evaluate the accuracy of model predictions. RMSE measures the average size of prediction errors and is particularly sensitive to large errors. MAE measures the average absolute value of errors and is generally more resistant to extreme values compared to RMSE. RMSLE, or Root Mean Squared Logarithmic Error, is a statistical metric employed in regression analysis to evaluate the accuracy of predictions. It emphasizes the relative difference between actual and expected values, rather than the absolute difference. The RMSLE value indicates a higher level of accuracy and better model performance. By utilizing these three criteria, one can evaluate the efficacy of the model through diverse methods, considering both its precision and suitability. The formulas for the three evaluation criteria are provided as follows:

$$\mathrm{RMSE}=\sqrt{\frac{1}{N}\sum_{i=1}^{N}{\left(y_{P,i}-y_{O,i}\right)}^{2}}$$ (14)

$$\mathrm{MAE}=\frac{1}{N}\sum_{i=1}^{N}|y_{P,i}-y_{O,i}|$$ (15)

$$RMSLE=\sqrt{\frac{1}{n}\sum_{i=1}^{n}{\left(log(y_{P,i}+1)-log(y_{O,i}+1)\right)}^{2}}$$ (16)

Here, *y*_*o*,*i*_ represents the observed run-off series, while *y*_*P*,*i*_ represents the predicted run-off series.

## 3. Results & discussions

The research investigation emphasizes the efficacy of advanced numerical weather prediction models in precisely forecasting average temperature, emphasizing the significance of real-time observational data in the forecasting process. The meticulous error investigation validates the models’ ability in accurately capturing long-term fluctuations in rainfall, humidity, and sea level pressure. Furthermore, this study introduces new approaches known as RNN-LSTM, TFDF and incorporates Stacking models. The effectiveness of these methods in predicting Temperature for the year 2024 is assessed by comparing them to existing data.

### 3.1 Meteorological analysis

Meteorological analysis is the methodical investigation of meteorological data to understand and predict weather patterns. The process involves a variety of techniques for observing and calculating weather conditions, such as surface and upper air observations, numerical weather prediction (NWP), climatic indicators, and forecasting. Surface observations provide data on temperature, humidity, wind speed, atmospheric pressure, and precipitation, while upper air observations provide information on temperature, pressure, and humidity. Climate indices aid in understanding global patterns and their influence on local weather. Meteorological analysis is crucial for understanding the Earth’s atmosphere, predicting weather events, and mitigating the impacts of extreme weather.

#### 3.1.1 Rainfall

**Fig 5:**
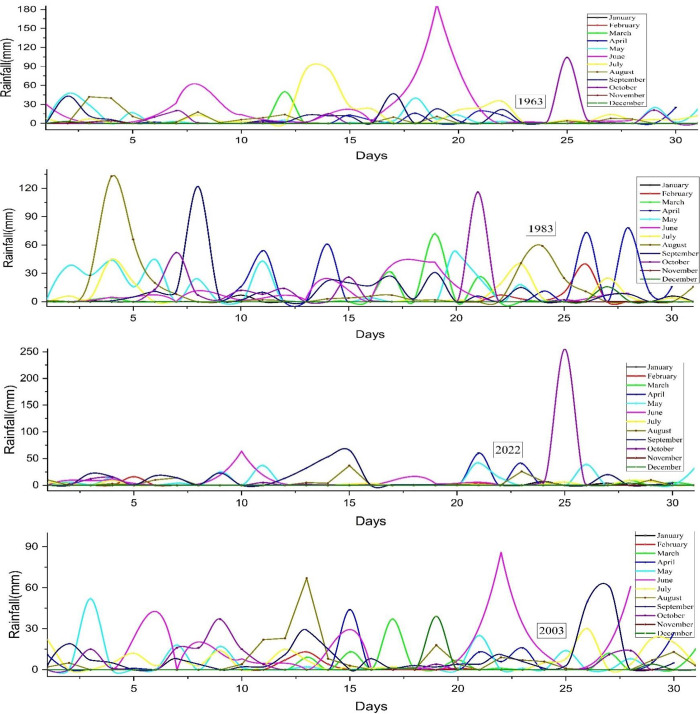
Rainfall distribution in Bangladesh, 1963–2022.

Rainfall is the downward movement of water droplets from the atmosphere to the Earth’s surface. It is crucial to maintain the balance of water around the globe. The occurrence of this phenomenon exhibits geographical variations influenced by a variety of meteorological and topographical factors. The factors that impact rainfall are the mean temperature, humidity, and sea level pressure. Dhaka, the capital of Bangladesh, experiences a monsoon-influenced tropical climate with distinct wet and dry seasons. The southwest monsoon is characterized by significant precipitation, while the dry season is distinguished by less rainfall. Dhaka is susceptible to tropical cyclones, especially during the monsoon season, which leads to substantial precipitation, strong winds, and flooding. Understanding regional precipitation patterns is essential for agricultural practices, water resource management, and urban development. **Fig 5** illustrates the fluctuation in rainfall across 20-year intervals spanning from 1963 to 2022. In June 1963, Bangladesh encountered a maximum precipitation of 180mm from the 16th to the 20th of that month. There was no rainfall fluctuation throughout September, October, November, and December due to a consistently minimal quantity of rainfall, approaching zero. Unlike 2003 and 2022, 1983 experienced substantial variations throughout several months, namely in August, September, and October. In 1984, a colossal flood occurred due to the significant amount of rainfall that occurred that year. The graph’s distribution in 2003 followed a normal distribution pattern, which was different from the distribution found in 1983. Nevertheless, in 2022, there was a substantial modification in the precipitation levels. The precipitation is reduced in comparison to the preceding 20-year interval. The cause can be ascribed to climate change.

#### 3.1.2 Humidity

**Fig 6:**
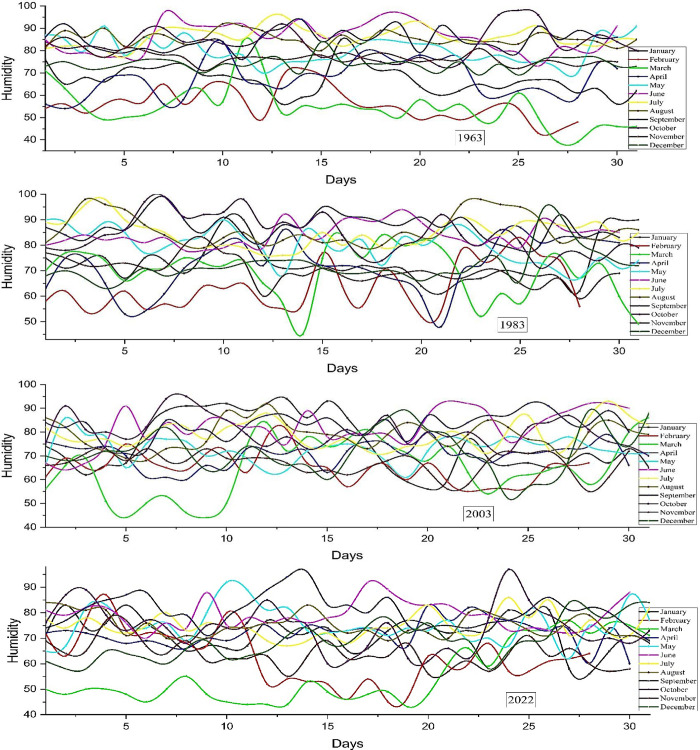
Humidity distribution in Bangladesh, 1963–2022.

Humidity, which refers to the amount of water vapor in the atmosphere, is crucial for Earth’s atmospheric conditions and significantly influences meteorological events, including rainfall. Humidity levels are determined by variables such as temperature, geographical location, and proximity to bodies of water. Geographical features also influence humidity, with coastal areas experiencing more humidity due to their proximity to large bodies of water, whereas inland locations often have lower humidity levels. Warmer seasons have increased humidity due to higher rates of evaporation. Humidity affects rainfall by promoting the process of condensation and the formation of clouds. Elevated levels of humidity facilitate the formation of clouds and precipitation, whereas reduced humidity can hinder cloud formation and reduce rainfall. Understanding variations in humidity is essential for predicting weather patterns and influencing ecosystems, agriculture, and water resource management. **Fig 6** illustrates the variation in humidity during a 20-year timeframe, from 1963 to 2022. In March 1963, there was a significant degree of volatility, characterized by a considerable decline in value towards the conclusion of the month. The weather is relatively cold in February, ranging from 40 to 70 degrees Fahrenheit. Nevertheless, it remains more desirable in comparison to the circumstances encountered in March. In late October, temperatures hit their maximum, nearing around 100 degrees. In the month of December, there is very little variation, with variations happening within a moderate range. In 1983, the patterns of 1963 were replicated, but they reach their lowest point in mid-March. However, throughout the month of August, it reaches its highest point between the 20th and 25th days. In 2003, the rate of change was relatively smaller compared to the preceding three years. In 2022, the months of September and October surpassed a threshold of 90%. The overall patterns have stayed almost unchanged for a duration of 60 years.

#### 3.1.3 Average temperature

**Fig 7:**
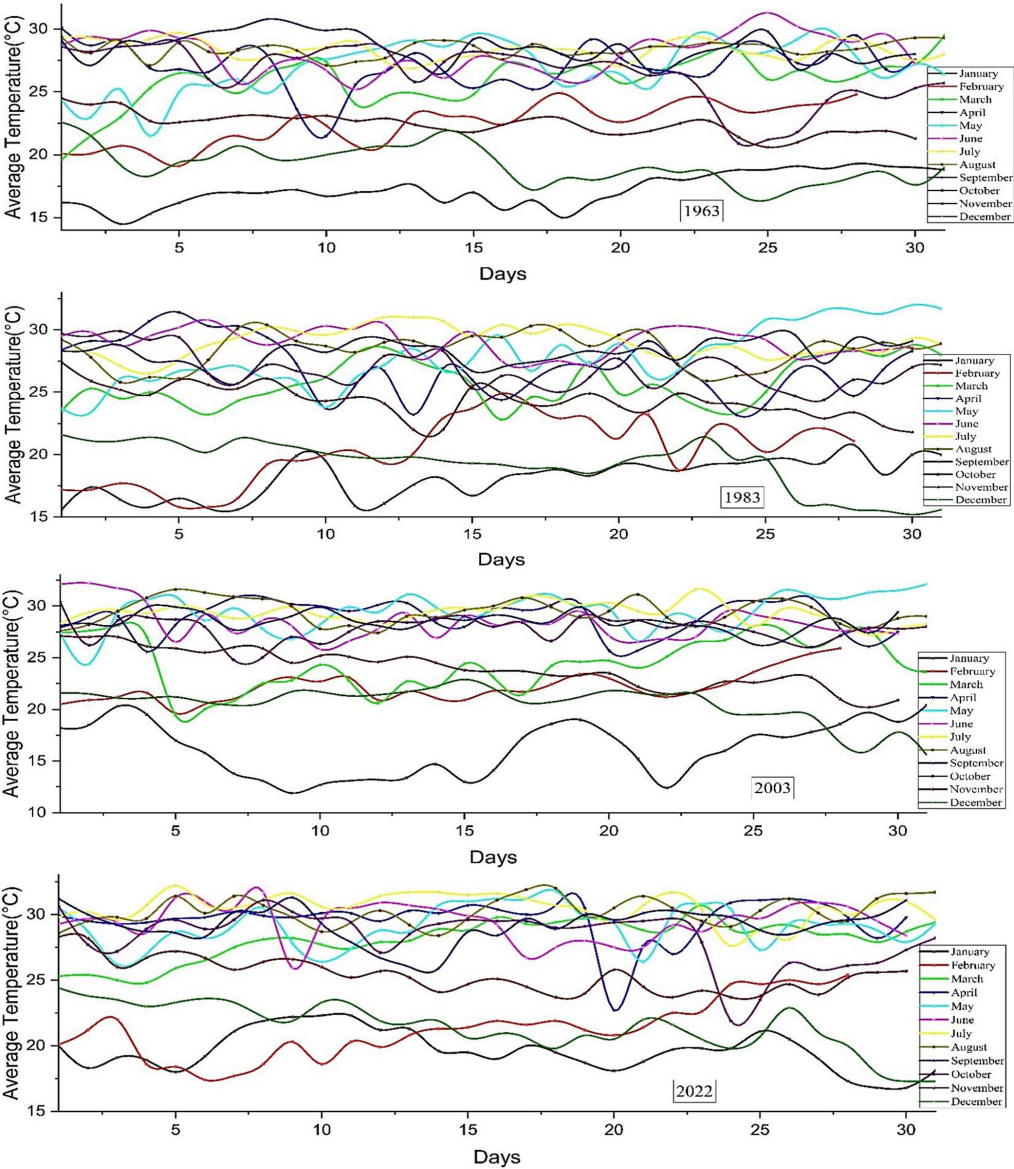
Average temperature distribution in Bangladesh, 2022.

The average temperature is calculated as the mathematical average of the temperature, considering factors such as latitude, altitude, and wind patterns. Changes influence the effect on rainfall in the water cycle, as greater temperatures promote increased evaporation and subsequent release of precipitation by clouds. Higher temperatures can hold more moisture, leading to a greater intensity of precipitation. Acquiring an understanding of common variations in temperature is crucial for predicting and understanding patterns in weather. By monitoring these variations, scientists can assess the impact of climate change on global weather patterns. According to **Fig 7**, the average temperature range in January was at its minimum in 1963, with temperatures ranging from 13° to 18°C. In September, the temperature peaked at 31°C and varied between 26° and 31°C. Nevertheless, the temperature reaches its highest point of the year in June, peaking at 32°C. The temperature hit its maximum at 34°C in 1983. In December, the temperature drops to a minimum of 15°C. In March, there are substantial and regular fluctuations in its levels or values. The temperature hit its maximum point at 37°C in May 2003. Extremely cold temperatures characterized the months of December and January in 2022. The temperature will not go below 17°C. The average temperature has increased compared to the previous 60 years. The data presented in the graph unequivocally demonstrates an increase in Bangladesh’s average temperature.

#### 3.1.4 Sea level pressure

**Fig 8:**
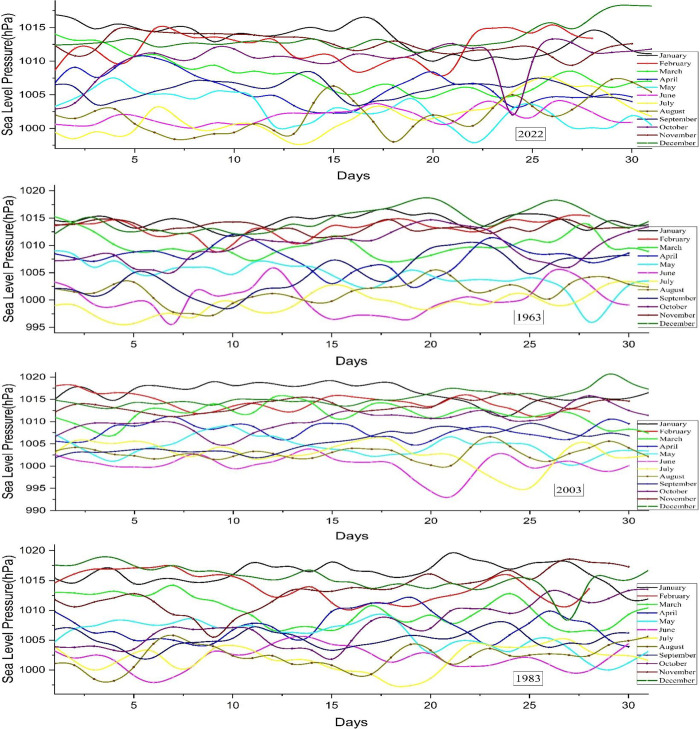
Sea level pressure in Bangladesh, 2022.

Sea level pressure refers to the atmospheric force exerted by the air above the Earth’s surface at sea level. As altitude increases, sea-level air pressure decreases, providing a dependable measure for comparing weather conditions. Pressure variations are associated with air masses and weather systems. High-pressure systems are responsible for steady weather patterns, while low-pressure systems cause turbulent weather, cloud formation, and precipitation. Monitoring sea level pressure patterns is crucial for weather forecasting as it provides crucial information on air circulation and the potential for precipitation. The maximum sea level pressure of 1017 hPa was recorded on December 20, 1963, surpassing the higher value reported in 1983, as depicted in **Fig 8**. The atmospheric pressure is 1019 hectopascals (hPa). Nevertheless, the highest recorded sea level pressure occurred in 2003, reaching a peak of 1022 hPa. The lowest temperature recorded in 2003 was 993 hPa, which occurred as part of a 60-year cycle. During June, the sea level pressure in May reaches its lowest point, which increases the likelihood of a cyclone.

### 3.2 Data processing & analysis

The normal probability plot is used to assess if a dataset conforms to a hypothesized distribution, with the vertical axis adjusted for a normal distribution. The Box-Cox transformation is applied to the distribution of the number of spaces to create sample frequency histogram and Q-Q plot.

The Q-Q plot displays the quantiles of a normal distribution against the quantiles of the data. The distribution of the number of spaces does not conform to a normal distribution. Q-Q plots and histograms are utilized to determine the necessity of transformation. For a right-skewed feature, a square root transformation is applied, while for a left-skewed feature, a squared transformation is utilized.

**Fig 9:**
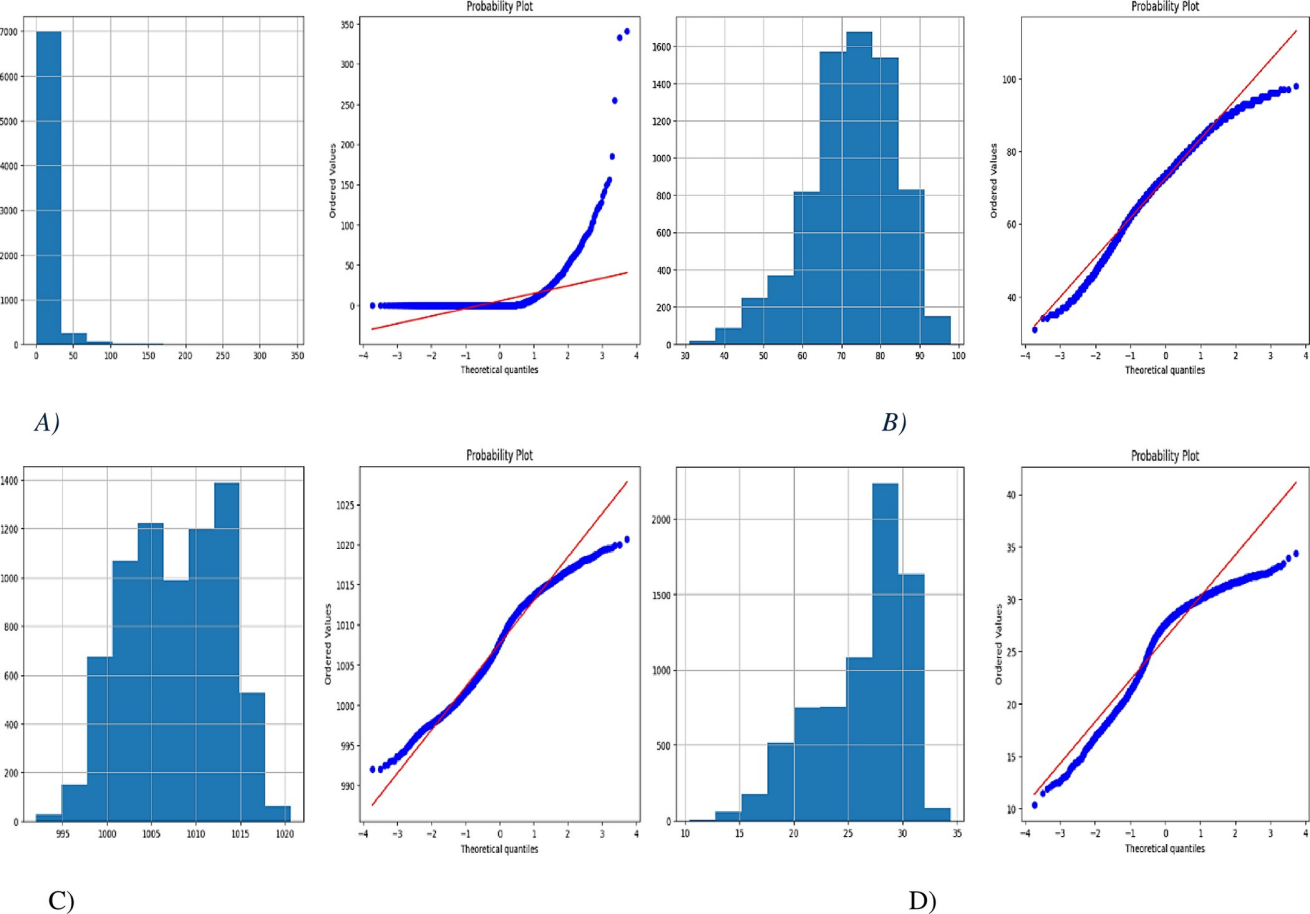
Histogram and Q-Q Plots of A) Rainfall B) Humidity C) Sea Level Pressure D) Average Temperature.

The probability plot of **Fig 9A** displayed a curvilinear distribution of points, whereas the histogram plot indicated a left-skewed variation. **Fig 9B** and **9C** are nearly identical. Histograms show central skewness, while the lines on the probability plot are nearly linear in the center. Both histograms showed no deviation from normality, indicating that the errors are independent. **Fig 9D** displays a right-skewed histogram. The transformation depicted in the histogram results in the ’Temp’ variable closely resembling a Gaussian distribution. The peak in most of the histogram is more centered, and the Q-Q plot shows deviations mainly at the extreme higher and lower values.

**Fig 10:**
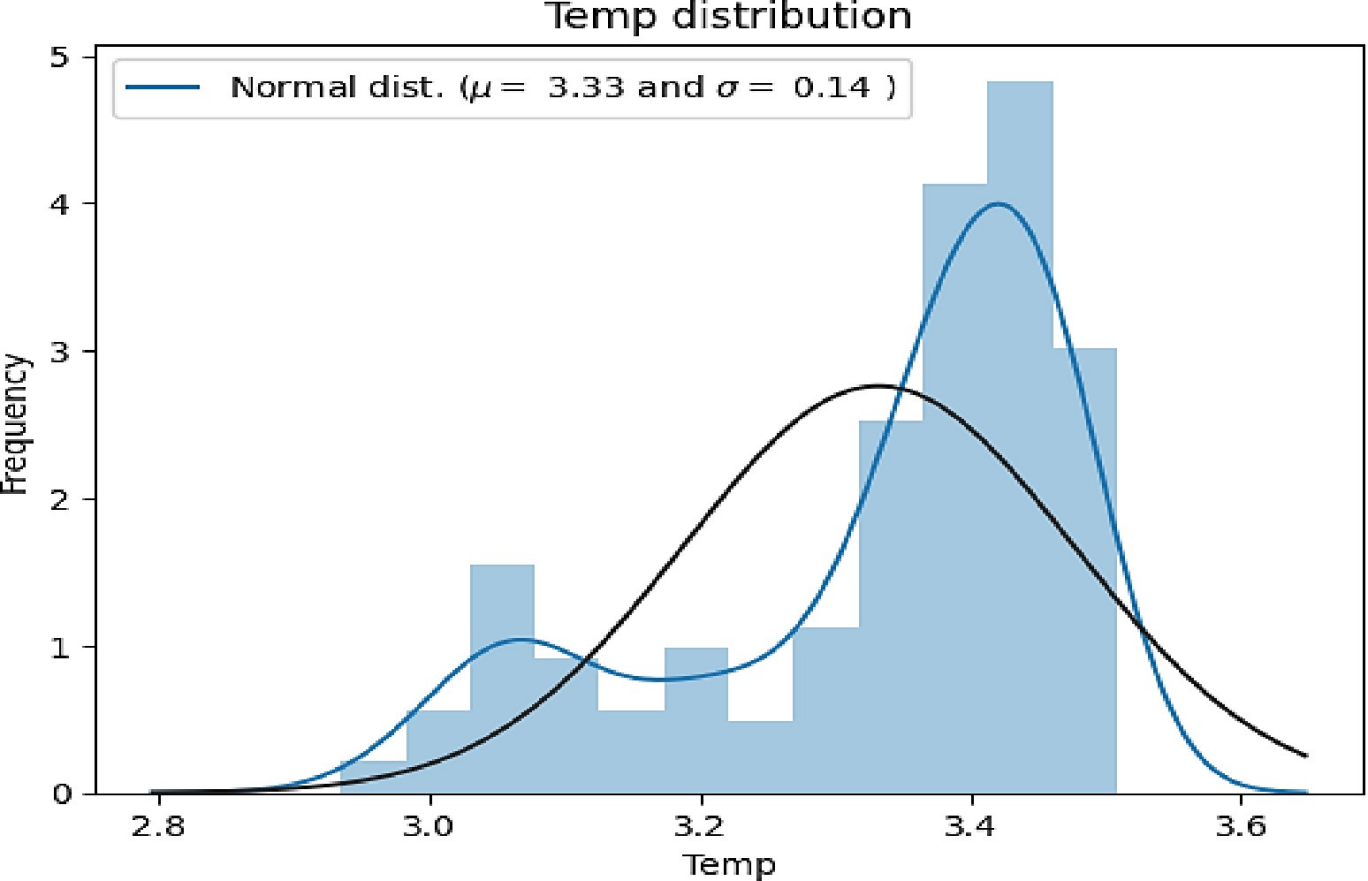
Normal probability plot of temperature.

**Fig 10** displays the standard normal distribution with a black line and the current distribution with a blue line. The mean is 3.33 while the standard deviation equals 0.14. There is a little change in the normal and current distribution. Non-linear transformation of the features is applicable when the Temperature data does not follow a normal distribution.

### 3.3 Sensitivity analysis

Sensitivity analysis is an essential technique in machine learning that assesses the impact of variations in input variables on a model’s predictions. The procedure entails evaluating the variability of data, color scales, identifying outliers, applying preprocessing techniques, selecting relevant features, determining resolution including bin size, managing noise, considering time series sensitivity, and analyzing interaction effects. The assessment examines the impact of data fluctuations on predictions, the influence of color scales on visualizations, and the effect of outliers on the model’s performance. Additionally, it analyzes the influence of various data resolutions on the precision of the model, its ability to handle noise, sensitivity to time series, and the effects of interactions on the model’s output.

Sensitivity analysis helps to understand heat maps, which are visual tools that depict the distribution and patterns of data within datasets. This comprehension enables making well-informed decisions on the interpretation and appropriate utilization of these visuals.

**Fig 11:**
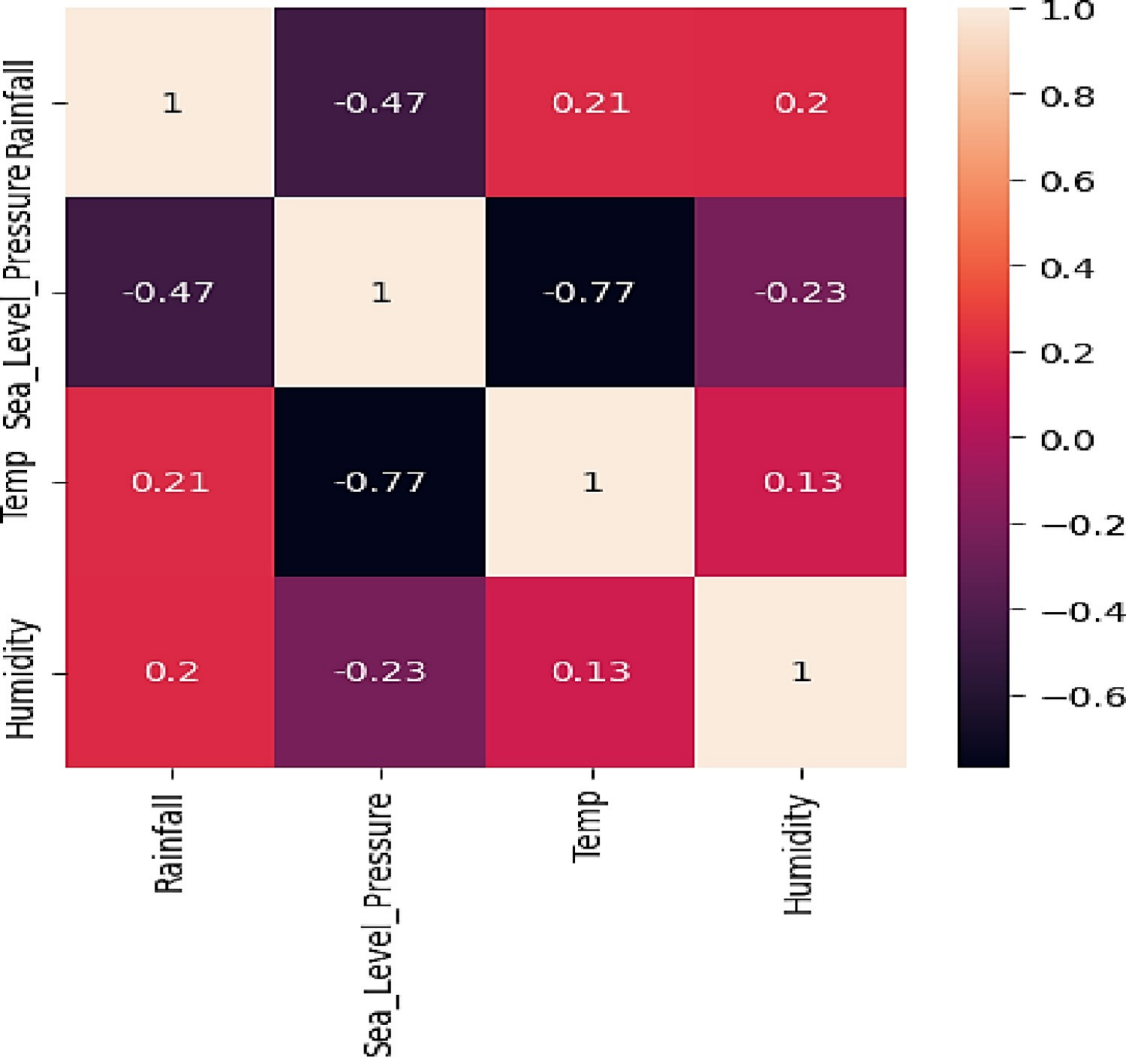
Analysis of meteorological data using heat map.

The **Fig 11** heat map illustrates how input parameters are related to each other and how sensitive they are to output parameters. The analysis demonstrates that every input parameter has an impact on the sensitivity of the outcome parameter, Rainfall. The study demonstrates that Sea Level Pressure has a detrimental effect on Rainfall, as evidenced by a correlation coefficient of -0.47, showing a reverse connection. The correlation values between humidity and all input parameters indicate a reasonable relationship, demonstrating that humidity has a balanced influence on rainfall. The Average Temperature exhibits a minor positive association with Rainfall, whereas it demonstrates a robust inverse correlation with both Average Temperature and Sea Level Pressure. The relationship between Rainfall and Average Temperature is characterized by an average positive relationship, as indicated by a correlation coefficient of 0.21. An evident contradiction exists between Average Temperature and Sea Level Pressure, with a correlation coefficient of -0.77.

### 3.4 Supervised learning model analysis

Supervised learning is a key principle in machine learning, involving training algorithms on labelled data to generate predictions or decisions. The process entails acquiring a mapping from input variables to output variables using example input-output pairs given in the training data. Evaluation measures include accuracy, precision, MAE, F1-score, RMSLE, RMSE and MSE for regression tasks. Supervised learning methods encounter obstacles, including overfitting, underfitting, data shortages, imbalanced datasets, and interpretability issues, regardless of their success. Supervised learning is widely used in healthcare, finance, marketing, and natural language processing. Comprehending the strengths, errors, and recommended procedures of various supervised learning models will assist professionals in constructing more precise and dependable predictive models to address intricate issues in various fields.

#### 3.4.1 RNN-LSTM

RNN-LSTM models are well-known for their capacity to recognize and make use of sequential relationships in input data. They are proficient in capturing extended relationships, managing sequences of varying lengths, acquiring temporal patterns, and surpassing constraints on short-term memory. Their gated architecture effectively addresses problems such as the vanishing gradient issue, leading to enhanced performance. RNN-LSTM models can effectively process sequences of different lengths while maintaining stable performance. They excel at acquiring temporal patterns, which are essential in activities where the sequence of elements greatly influences the result. The models excel in tasks with sequential data by preserving information across numerous time steps, a capability lacking in classic feedforward neural networks. Nevertheless, they can also involve computational costs, particularly when handling extensive datasets.

**Fig 12:**
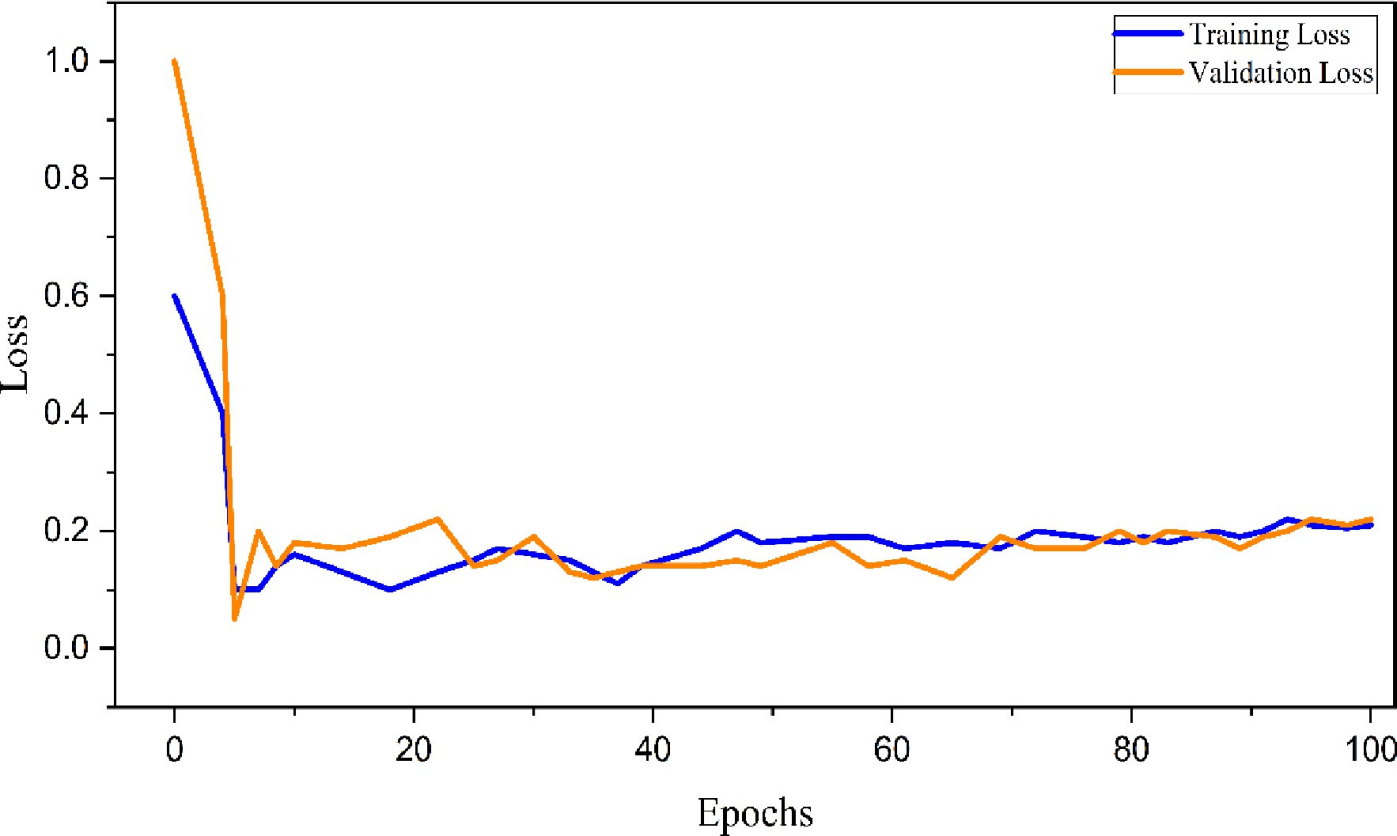
Training and validation loss of RNN-LSTM model.

The loss function, training loss, and validation loss are vital instruments in machine learning, offering valuable insights into a model’s performance throughout the training process. The loss function quantifies the discrepancy between the anticipated output and the true ground truth, with the training loss indicating the error computed on the training dataset. Validation loss is computed on a distinct dataset, whereas overfitting happens when the model becomes overly intricate and starts to capture noise or random variations. Methods such as regularization, dropout, and early stopping can assist in reducing overfitting. Ideally, both training and validation losses should decrease concurrently, demonstrating successful learning without overfitting. **Fig 12** illustrates the lack of overfitting. After 30 epochs, the learning process has stabilized and indicated a drop in both training and validation loss.

**Table 1** displays the outcomes of the experiments conducted on architectures with 500 and 1000 nodes. The process of optimizing a neural network involves the measurement of loss. The RNN-LSTM model achieved a loss of 163.52 and a mean edit distance of 416.41when trained on 500 nodes. However, while considering 1000 nodes, RNN-LSTM obtained a score of 145.672 and a mean edit distance of 308.996. Regarding the RMSLE, the 1000-node had a significant impact, with a value of 1.430. It was observed that 1000 nodes yielded the highest performance.

**Table 1:** Results for RNN-LSTM layer architecture.

Period	Algorithm	Loss	Mean Edit Distance		RMSLE
500-node Layer	RNN-LSTM	163.52		416.41	1.453
1000-node Layer	RNN-LSTM	145.672		308.996	1.430

**Fig 13:**
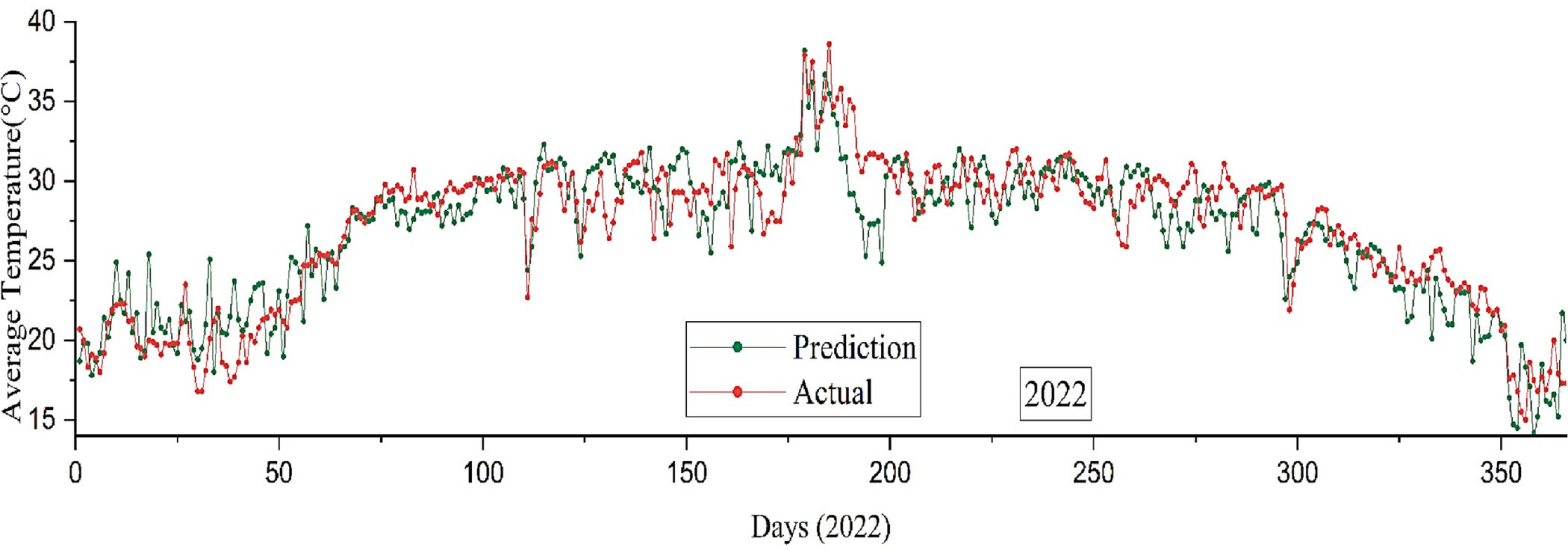
Average temperature forecast by the RNN-LSTM model versus the actual value in 2022.

The present study utilized the RNN-LSTM model to generate an accurate forecast of the average temperature in Bangladesh during the calendar year 2022. The accuracy of the prediction model is depicted in **Fig 13**, indicating a moderate level of precision at 74%. In order to validate the result, empirical data from 2022 has been used. The observed data is denoted by the red line, whereas the prediction data for a duration of 365 days is represented by the green line.

#### 3.4.2 TFDF

**Fig 14:**
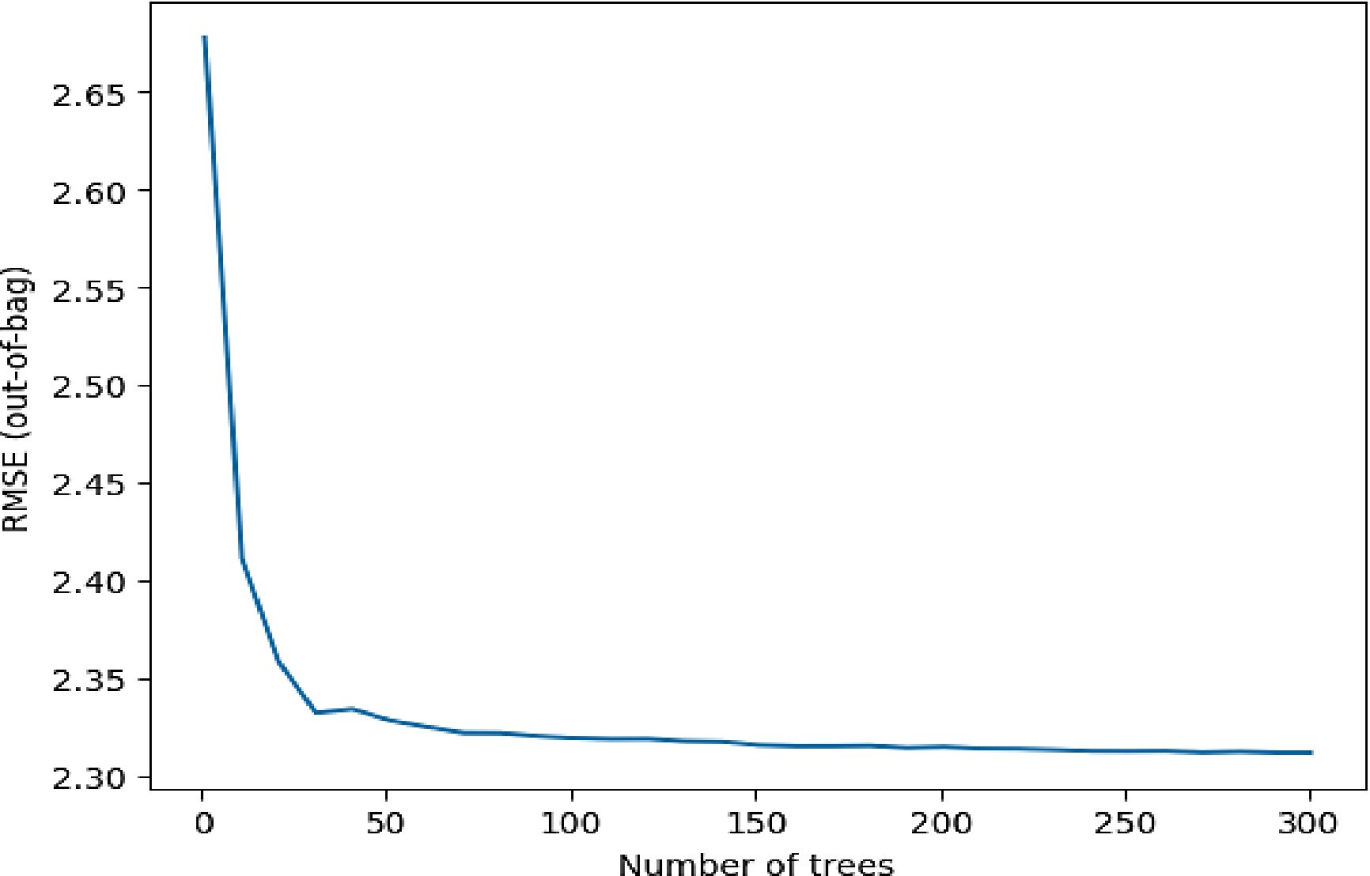
Graph illustrating prediction error against the number of trees.

**Fig 14** shows the relationship between RMSE and the number of trees in TensorFlow Decision, which offers an understanding of how the model’s performance changes as model complexity increases. The x-axis depicts the quantity of trees in the ensemble, usually starting at a small amount and progressively increasing from 0 to 300 trees. The y-axis depicts the model’s prediction inaccuracy on data that has not been seen before. Initially, there is an inverse relationship between the number of trees and the RMSE (out of bag), with the RMSE decreasing as the number of trees increases starting at 2.70 RMSE value. This indicates that the ensemble method is effective in capturing intricate patterns and minimizing prediction mistakes. The saturation point is 2.33, indicating that further tree additions are unlikely to substantially enhance predictive performance. The ideal number of trees is typically found close to this point, achieving a balance between model complexity and forecast accuracy.

**Fig 15:**
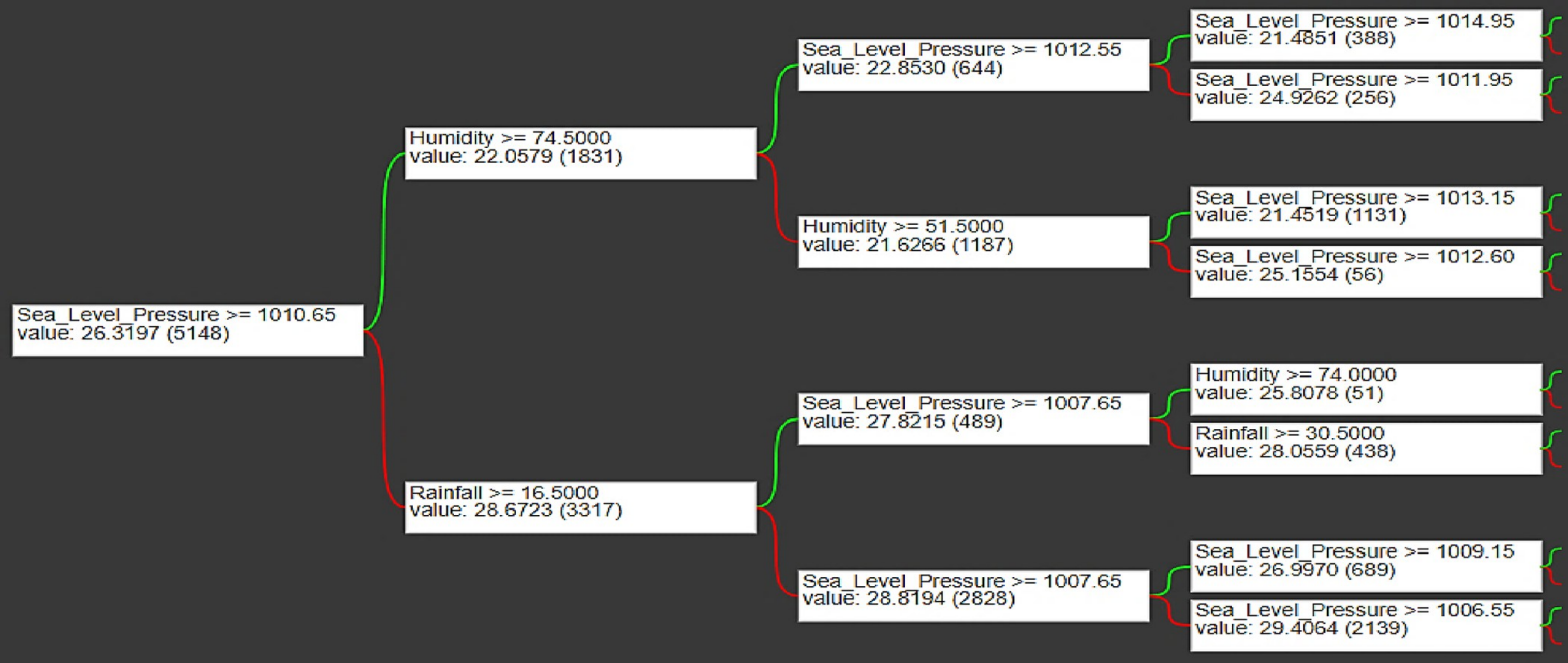
Construction of a TensorFlow decision forest plot model.

TensorFlow Decision Forests does not include an integrated "plot_model" method to show the model’s overall structure, however it does allow visualization of individual trees with feature importance. The plot_tree function illustrates a decision tree graphically, while the plot_feature_importance function displays the significance of several characteristics. **Fig 15** shows a tree plot with a maximum depth of 3, along with an explanation of the parametric range. The tree structure is visually traceable. The initial decision in this tree is determined by the Sea Level Pressure. When the Sea Level Pressure is below 1010.65 hPa, it is likely to start a weather prediction procedure shown by green and red lines.

**Fig 16:**
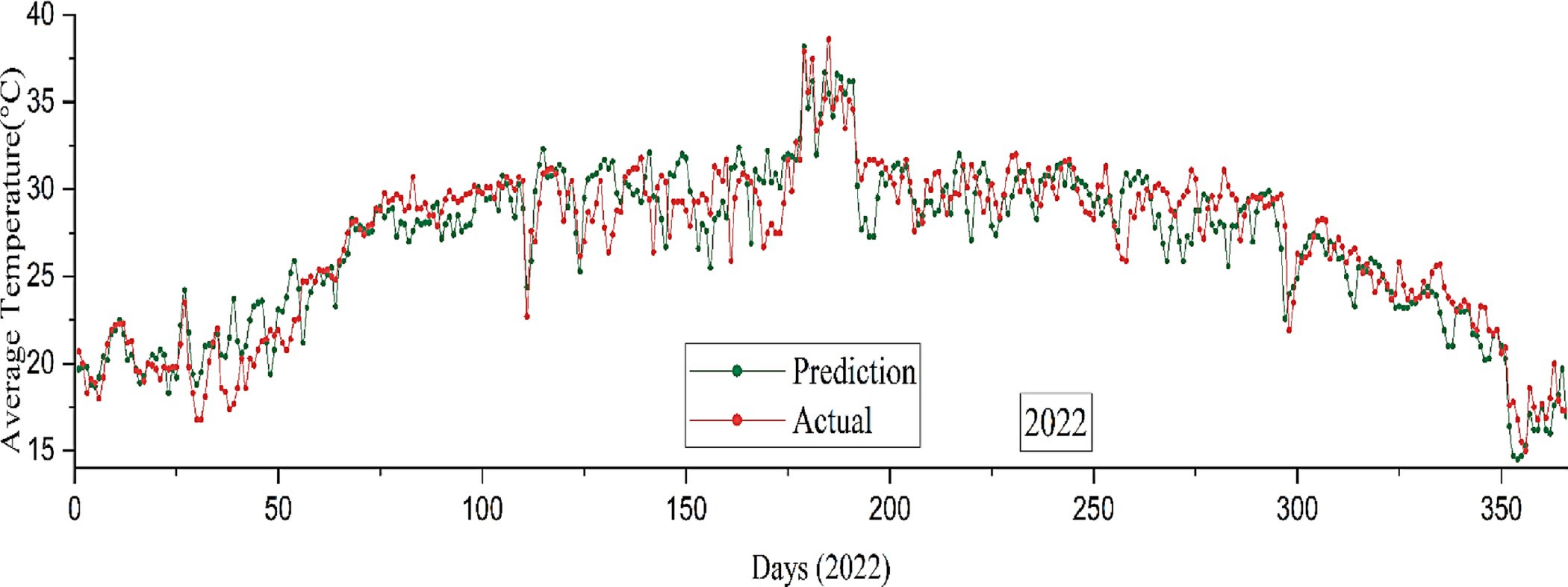
Average temperature forecast by the TFDF model versus the actual value in 2022.

The study conducted a comparison between the performance of TensorFlow Decision Forest and several error analysis methods. **Table 2** presents the error analysis of the validation and testing data, and also provides a comparison of the results. The validation metrics for MAE, RMSE, and RMSLE are 157.448, 419.3287, and 1.397, respectively. However, upon examining the testing data, it reveals the values 198.531, 502.459, and 1.442. The testing data values are higher than the validation data values due to the random selection of data for testing the model. Furthermore, the forecast results show a moderate level of precision, with an accuracy of 82.1%, as depicted in **Fig 16**. While this accuracy is not as high as that of the stacking model, it still outperforms the RNN-LSTM model, demonstrating a reasonable ability to capture the data’s nuances.

**Table 2:** Evaluation criteria for TFDF model.

Period	Algorithm	MAE	RMSE		RMSLE
Validation	TFDF	157.448		419.3287	1.397
Testing	TFDF	198.531		502.459	1.442

#### 3.4.3 Stacking model

The stacked averaged models achieved an RMSLE score of 1.3002, which is higher to the score of every other base model and also surpasses the results of the separate stacking models.

The models were evaluated according to their performance, and the assessment results for all three of them during the validation and testing periods are presented in **Table 3**. Each of the three base models demonstrates differences in their MAE, RMSE and RMSLE. **Table 3** shows that the Elastic Net model displays the lowest overall performance compared to the other models.

**Table 3:** Criteria for evaluating three basic models.

Period	Algorithm	MAE	RMSE	RMSLE
Validation	Elastic Net	242.733	544.124	1.4122
	GradiantBoost	212.335	502.228	1.3689
	KRR	202.388	389.455	1.3301
Testing	Elastic Net	253.818	636.173	1.4356
	GradiantBoost	242.167	502.934	1.39
	KRR	204.389	384.337	1.3445

Throughout the validation and testing phase, the KRR model exhibited superior performance compared to the Elastic net and Gradient Boosting models, which were employed to represent the machine learning ensemble model. The KRR model includes an RMSE of 384.337, an MAE of 204.389, and an RMSLE of 1.3445. Among the three models, the KRR model exhibits the best performance across three measures, with a notable improvement of 24.1838% along with 18.4834% in MAE relative to the Elastic net as well as GradientBoost models, respectively. In addition, the KRR model offers the lowest standard deviations for each of the criteria, indicating a high degree of stability in its performance.

The study conducted a comparative analysis of the performance of the basic models and the stacked models. **Table 4** presents the evaluation criteria for the three stacking algorithms, including two model forecasts throughout the testing stage in the study area. The optimal baseline model has an RMSE of 384.337, an MAE of 204.389, and an RMSLE of 1.4963, as depicted in **Tables 3** and **4**. On the other hand, the SAE model displays a RMSE of 403.56, MAE of 232.55, with RMSLE of 1.4963. The SAE model, which is the simplest stacking model, clearly failed to outperform the performance of the ideal base model. However, it demonstrates superior outcomes in comparison to other foundational models. The SAE model’s prediction was obtained by computing the weighted mean of all the forecasts generated by the base models. The operational efficiency of the SAE model is generally moderate. The two remaining stacking models surpassed the base models in all respects. According to the statistics, the ATE model performs better than the KRR model, showing an improvement of 29.848% for RMSE, 9.934% for MAE, and 1.50234% for RMSLE.

**Table 4:** Evaluating the stacking prediction model.

Models	RMSE	MAE	RMSLE
SAE	403.56	232.55	1.4963
WAE	334.85	208.61	1.3718
ATE	295.99	185.92	1.3246
LightGBM	201.43	131.08	1.2269
XGBoost	165.82	177.11	1.3823

The WAE model indicated similar improvements to those of the base models. By integrating machine learning models with diverse topologies, the stacking model has the ability to surpass the learning efficiency of its underlying models.

### 3.5 Stacked model equation

**Stacked averaged models** = Stacked_ average_models [*Base_models = (Elastic_Net*, *Gradiant_Boost*, *KRR)*, *meta_model = lasso_model)*]

**Predictions** = stacked_predictions*0.60 + xgboost_predictions*0.20 + lgbm_predictions*0.*20*

**Fig 17:**
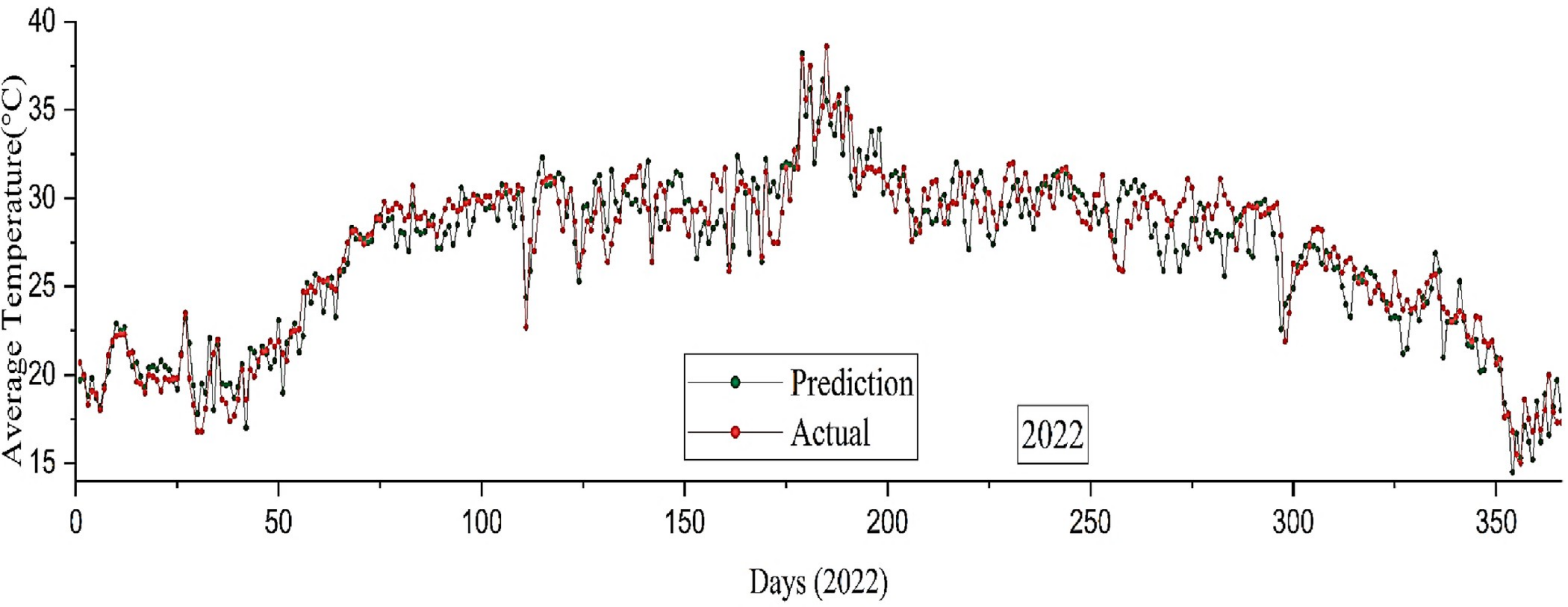
Average temperature forecast by the Stacking model versus the actual value in 2022.

After executing the ensemble model with XGBoost along with LightGBM, the achieved RMSLE score is 0.8235, representing the highest among all models. Furthermore, the forecast result exhibits a notable degree of precision, measuring at 91.5% as illustrated in **Fig 17**. The graph displays a complete projection for the year 2022 in Bangladesh, calculated using a stacked model.

### 3.6. Future prediction

**Fig 18:**
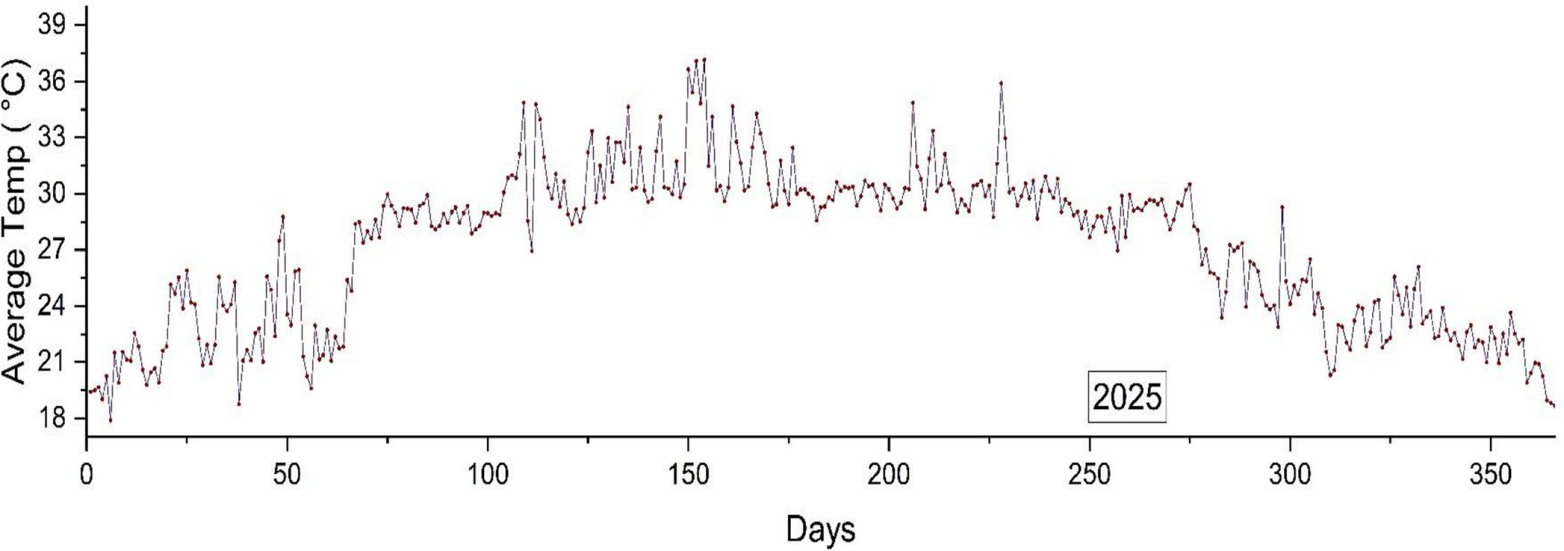
Future average temperature prediction for Bangladesh (2025).

The results of this study showed that the stacked model outperformed other algorithms. Therefore, the model generates the graph in **Fig 18**, which forecasts future results. The graph displays the anticipated mean temperatures for Bangladesh in 2025, illustrating a persistent pattern of increasing heat over an extended period of time. Distinct seasonal fluctuations are evident, with the summer months exhibiting elevated temperatures in comparison to the previous year. The anticipated rise in temperatures may result in significant environmental consequences, such as changes in ecosystems, the loss of habitats, and changes in the pattern of precipitation. Aside from problems with health, rising temperatures may also have an effect on the economy and agriculture. The limitations of the prediction model encompass uncertainty in climate models, anticipated fluctuations in greenhouse gas emissions, and regional climate dynamics. Climate models are essential tools for simulating the Earth’s climate system and forecasting future climatic conditions. Nevertheless, uncertainties emerge due to various reasons including model structure, boundary conditions, internal variability, forcing scenarios, and greenhouse gas emissions. The presence of these uncertainties might result in discrepancies in temperature predictions and a lack of predictability. High emission scenarios lead to more pronounced global warming, because greenhouse gas emissions are the primary driver of climate change. In order to improve the reliability of temperature predictions, it is crucial to include the uncertainty stemming from climate models and greenhouse gas emissions into machine learning algorithms. Ensemble modeling, employing a compilation of climate models, can provide a range of possible outcomes, assisting in the depiction of uncertainty. Ensemble learning techniques, such as bagging and boosting, enhance the dependability of machine learning predictions by combining many models. Probabilistic forecasting use machine learning techniques to generate a spectrum of likely temperature outcomes and their corresponding probabilities, enabling more precise assessment of potential risks and informed decision-making. Scenario analysis aids policymakers and stakeholders in comprehending the potential ramifications of various emission reduction strategies and their effectiveness in avoiding temperature rise. Uncertainty quantification tools, such as Monte Carlo simulations, Bayesian inference, and sensitivity analysis, can assess and analyze the level of uncertainty in machine learning predictions.

## 4. Conclusions

An investigation on the meteorological study of Bangladesh spanning the years 1963 to 2022 has been carried out. The primary goal was to forecast the average temperature and its variations over a 60-year period using a parametric study. The level of average temperature undergoes a swift and substantial increase as a result of the influence of humidity. During the month of April, there was a substantial heat generated. The drop in sea level pressure coincides with the increase in average temperature, resulting in heightened rainfall also the humidity. This work uses the latest algorithm RNN-LSTM, TFDF and a novel technique dubbed the Stacking model to predict meteorological features, taking into account the non-linear tendencies of the weather. Based on the research, the subsequent conclusions can be inferred:

- An analysis of meteorological data in Bangladesh over the past sixty years indicates notable alterations in atmospheric factors, specifically the average temperature, from 2003 to 2022. These changes have been linked to climate change, which has disrupted the usual weather patterns and led to drought conditions in the 20th century.
- The Stacked average model shows the highest level of precision, as seen by its rmsle value of 1.3002. Yet, it necessitates an extensive time of training and a substantial amount of memory. The individual base as well as meta models exhibit diminished accuracy but require a shorter duration for training. Hence, it is recommended that the suggested mean model be utilized for real-world implementations.
- The TFDF model indicates a 0.698% higher precision in comparison to the RNN-LSTM model, however, the error outcomes of the stacked average model exceed those of both models.
- Based on the stacked forecasting model, it is clear that the ATE model performs better than the SAE model, with a 12.9625% improvement in the prediction process. The RMSE value for the WAE is 334.85, making it superior to the SAE but inferior to the ATE.

To improve future analysis, it is advisable to include additional dependent factors pertaining to climate patterns, especially wind speed, dew point, and GHI, in the prediction. This research can be applied in CSP-based sCO_2_ power plants or smart sensors utilized in agriculture. By integrating DNN with imaging techniques, it is possible to perform a comparative analysis to distinguish the differences between numerical approaches and neural networks. The final objective is to strengthen the ability to forecast.
